# Family influences on child nutritional outcomes in Nairobi's informal settlements

**DOI:** 10.1111/cch.12670

**Published:** 2019-05-20

**Authors:** Cheikh Mbacké Faye, Sharon Fonn, Elizabeth Kimani‐Murage

**Affiliations:** ^1^ Research Division African Population and Health Research Center Nairobi Kenya; ^2^ School of Public Health University of the Witwatersrand Johannesburg South Africa

**Keywords:** child feeding, exclusive breastfeeding, grandmothers, malnutrition, Nairobi, stunting

## Abstract

**Background:**

Improving child nutritional status is an important step towards achieving the Sustainable Development Goals 2 and 3 in developing countries. Most child nutrition interventions in these countries remain variably effective because the strategies often target the child's mother/caregiver and give limited attention to other household members. Quantitative studies have identified individual level factors, such as mother and child attributes, influencing child nutritional outcomes.

**Methods:**

We used a qualitative approach to explore the influence of household members on child feeding, in particular, the roles of grandmothers and fathers, in two Nairobi informal settlements. Using in‐depth interviews, we collected data from mothers of under‐five children, grandmothers, and fathers from the same households.

**Results:**

Our findings illustrate that poverty is a root cause of poor nutrition. We found that mothers are not the sole decision makers within the household regarding the feeding of their children, as grandmothers appear to play key roles. Even in urban informal settlements, three‐generation households exist and must be taken into account. Fathers, however, are described as providers of food and are rarely involved in decision making around child feeding. Lastly, we illustrate that promotion of exclusive breastfeeding for 6 months, as recommended by the World Health Organization, is hard to achieve in this community.

**Conclusions:**

These findings call for a more holistic and inclusive approach for tackling suboptimal feeding in these communities by addressing poverty, targeting both mothers and grandmothers in child nutrition strategies, and promoting environments that support improved feeding practices such as home‐based support for breastfeeding and other baby‐friendly initiatives.

**Key messages**

- Grandmothers have central roles in child nutrition as they are key advisors to younger mothers in addition to being child caregivers themselves. They are important targets for child health interventions and should not be undermined in strategies aiming to reduce child malnutrition in urban informal settings.
- Child health interventions in urban informal settings not only should focus on increasing mothers' knowledge on child nutrition but also should include promotion of optimal child‐feeding practices and promotion of interventions that support mothers to combine breastfeeding and their other occupations.
- Reinforcement of interventions towards sustainable poverty reduction is fundamental in dealing with child undernutrition.

## 1 INTRODUCTION

Nutrition is one of the most important factors that impact on children's development and is linked to short‐ and long‐term cognitive development and survival (Grantham‐McGregor et al., 2007; Oddy et al., 2003; Victora et al., 2008). Severely stunted children face a four times higher risk of dying by 5 years than normal children (Black et al., 2008). Stunted children enter adulthood with a greater propensity for developing obesity and chronic diseases (Hoffman, Sawaya, Verreschi, Tucker, & Roberts, 2000; United Nations Children's Fund [UNICEF], 2013). In developing countries, particularly in sub‐Saharan Africa, the prevalence of child stunting remains high. In 2016, it was estimated that 34% of children under‐five years in sub‐Saharan Africa were stunted, Eastern Africa being the most affected African region, where the prevalence was 37% (UNICEF—World Health Organization [WHO] and The World Bank, 2017). Unless stunting can be reversed, achieving the Sustainable Development Goals, in particular, Sustainable Development Goals 2 and 3, which aim respectively to end hunger and promote good health and well‐being for all at all ages, seems unlikely.

In Kenya, stunting prevalence among under‐five children remained high at 26% in 2016 (UNICEF—WHO and The World Bank, 2017). However, this national figure conceals intracountry differences. The prevalence of stunting in Nairobi informal settlements was close to 50% among children under 5 years (Kimani‐Murage et al., 2015) and has been reported to be 60% among children by the age of 15 months during the period 2006 to 2010 (Fotso et al., 2012). As in many African cities, Nairobi is urbanizing quickly (Chirisa, 2008; Obudho & Aduwo, 1992) with a large proportion of the urban population living in informal settlements within or on the periphery of the city (Zulu, Dodoo, & Ezeh, 2004). These settings are mainly characterized by poor living conditions including overcrowding, high poverty levels and fertility rates, and poor access to health care services (Emina et al., 2011), which expose young children to health hazards and a heightened risk of morbidity and mortality (African Population and Health Research Center [APHRC], 2014; Fotso, 2007).

**Figure 1:**
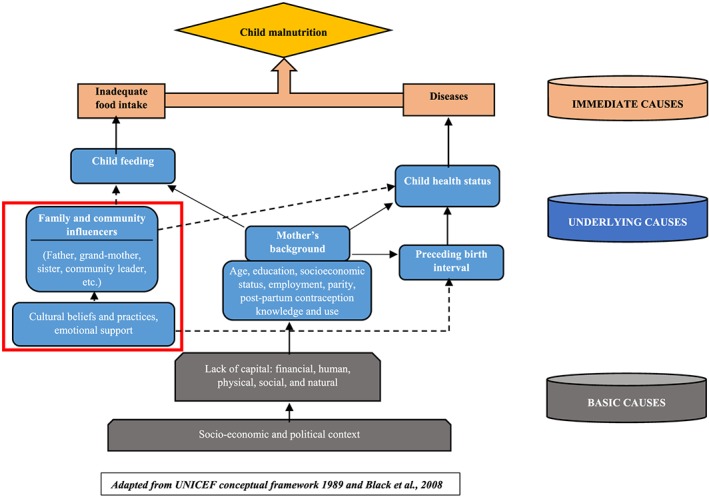
Conceptual framework of factors influencing child nutrition [Colour figure can be viewed at wileyonlinelibrary.com]

The burden of child stunting in Nairobi slums has been documented (Abuya, Ciera, & Kimani‐Murage, 2012; Fotso et al., 2012; Kimani‐Murage et al., 2015), and some interventions have been attempted in the last few years to address child health issues, including nutrition, in deprived areas in the city (Amendah, Mutua, Kyobutungi, Buliva, & Bellows, 2013; Bakibinga et al., 2014; Kimani‐Murage et al., 2016). Nonetheless, child stunting remains a public health problem in these settings. This may indicate that the complexity of interrelated causes of stunting is not sufficiently understood to assist in developing more successful interventions. Although social determinants of child nutrition, including stunting, in similar settings have been investigated, most of the studies emphasized individual level factors such as mother and child attributes (Abuya et al., 2012; Kandpal & McNamara, 2009; Kimani‐Murage et al., 2015; Olack et al., 2011). Less is known about household and community level factors underlying individual behaviours and practices. Some studies highlighted community level factors that may influence child nutrition, such as cultural and religious beliefs (Goudet et al., 2017; Kimani‐Murage et al., 2014; Wanjohi et al., 2017). It is at household and community levels where hierarchical authority and informal communication networks operate and may influence child nutrition practices. Furthermore, the role and influence of indirect factors, such as fathers and grandmothers, have received limited attention. In a previous quantitative study in the same settings, we reported significant household random effects among the determinants of child linear growth (Faye, Fonn, Levin, & Kimani‐Murage, 2019), suggesting the presence of underlying household level factors. Building on the current literature and UNICEF framework on child health (UNICEF/WHO, 1989), we extended the intermediate causes of child malnutrition by including family and community influence as social determinants of child nutrition (Figure 1, red square) to shed more light on household level decision making. The influence of household members on child feeding in two informal settlements in Nairobi—what to give the child and when—is explored in this study.

The composition of households and characteristics of the head of household are important factors affecting household welfare (Tai & Treas, 2009). In Kenya, the mean size of households is 4 (3.2 urban vs. 4.4 rural) and about two‐thirds of the households are male headed (Kenya National Bureau of Statistics, 2015). In the study settings where this research was done, the average number of household members is below the national level and varies between 2.5 in Korogocho and 2.9 in Viwandani (African Population and Health Research Center, 2017). Looking at household headship, eight households out of 10 in the two sites are male headed (African Population and Health Research Center, 2017). In similar African informal settings, households are mostly structured around a couple (husband and wife), a child, and a fourth person who is usually a parent to the couple (Collette, 1993; Ekane, 2013). Decision‐making mechanisms in such extended families, including on child health and feeding, are not necessarily under the sole control of the mother or caregiver. In many cases, the voice of the oldest household member or that of the mother's closest confidant within the household is highly valued (Ekane, 2013; Therborn, 2006). Cultural beliefs and practices reinforced by family members have been shown to influence mothers' decisions related to child feeding in developing countries (Aubel, 2012; Daglas & Antoniou, 2012). Extended families may still have a hold in many African countries despite urbanization. In this study, we explored the roles of family members, in particular, grandmothers and fathers who are living with the mother, in decision making pertaining to child feeding.

## 2 METHODS

### 2.1 Study settings

The study was conducted in two Nairobi informal settlements: Korogocho and Viwandani. The sites are located approximately 10 km from the city centre and about 7 km from each other (Emina et al., 2011). Each occupies about 0.5 km^2^ and is densely populated: 30,736 residents in Korogocho and 52,852 residents in Viwandani (African Population and Health Research Center, 2017). The sites are characterized by high unemployment, poverty, and poor access to facilities such as water, sanitation, and health care services when compared with Nairobi as a whole (APHRC, 2002). However, there are few differences between the two sites. For instance, Viwandani is bordered by an industrial area and attracts a youthful and highly mobile population, whereas in Korogocho, the population is more stable, about a quarter of the residents aged 12 years and above were born in this informal settlement (Emina et al., 2011). A variety of ethnic groups live in the sites, specifically *Kikuyu*, *Luhya*, *Luo*, *Kamba*, and *Somali*, with different cultural norms and practices with regard to child development including feeding.

### 2.2 Study participants and data collection

The study targeted mothers of children aged 12–59 months and family influencers such as grandmothers and fathers. The participants were selected purposively from a 2006–2013 Maternal and Child Health project nested within the Nairobi Urban Health and Demographic Surveillance System (NUHDSS). The NUHDSS collects birth, death, and migration data every 4 months, and the Maternal and Child Health study collected child anthropometric measurements. From these data, households with stunted and not stunted under‐five children were identified. Community health workers in the study area helped identify the selected households based on their NUHDSS unique identifier. In total, 30 in‐depth interviews were conducted in the two sites: 12 with mothers of stunted children, eight with mothers of nonstunted children, six with grandmothers, and four with fathers, as shown in Table 1. Respondents were interviewed if they met three inclusion criteria: (a) mothers, grandmothers, and fathers of at least one child aged 12–59 months present at the time of interview, (b) living in one of the study sites since birth of the last child, and (c) willing to participate in the study. In most cases, mother, father, and grandmother from the same household were interviewed. Data collection took place in September 2016 using three different interview guides (one each for mothers, grandmothers, and fathers). The study collected information on respondents' knowledge on child stunting, practices and beliefs on child feeding, and child health care. The interviews were all conducted in Kiswahili by two experienced research assistants after intensive training by the study principal investigator into the purpose of the study and the instruments to be used. The interview guides were pretested on respondents with similar background to the study participants. Field supervision was done by the principal investigator.

**Table 1:** Number of in‐depth interviews by type of respondent and study site

Respondents	Viwandani	Korogocho	Total
Mothers of stunted children	6	6	12
Mothers of nonstunted children	4	4	8
Grandmothers	3	3	6
Fathers	2	2	4
Total	15	15	30

### 2.3 Data analysis

The interviews were audio‐recorded then transcribed before translation into English. Using a deductive coding approach, the analysis was done using Nvivo 10 and guided by the conceptual framework on child health described in Figure 1 to explore the household level factors underlying child health and feeding practices. The coding approach was informed by findings from previous research conducted on child growth in the same settings, in particular, household level factors that were related to child linear growth (Faye et al., 2019). We explored respondents' knowledge on child stunting (perceptions and perceived causes), practices around child feeding in the household, decision making on child feeding (who decides on what the child should eat and when), and child health and care (what to do when a child is identified or perceived to be stunted and who takes the decision). Consistency checks were applied during the analysis and coding to ensure good understanding and interpretation of the data. The consistency checks consisted of triangulation (utilizing two analysts to review the data and findings) and member checking where each respondent was given a chance to confirm his or her views and statements before leaving the interview venue. Thematic analysis was used to make sense of the data (Grbich, 2012; Vaismoradi, Turunen, & Bondas, 2013).

### 2.4 Ethics

The study was approved by the African Medical and Research Foundation Ethics and Scientific Review Committee in Kenya and the Human Research Ethics Committee (Medical) at the University of Witwatersrand in South Africa. All interviews were conducted in private, and written informed consent was sought from all participants.

## 3 RESULTS

Table 2 shows the characteristics of respondents. The average age was 43 years (mothers 32, grandmothers 52, and fathers 49). Some diversity was noted among mothers in terms of educational level, ethnicity, and parity.

**Table 2:** Background characteristics of the respondents

Background characteristics	Mothers (n = 20)	Grandmothers (n = 6)	Fathers (n = 4)	Total (n = 30)
Site				
Korogocho	10	3	2	15
Viwandani	10	3	2	15
Mean age (years)	32	52	49	43
Ethnic group				
Kikuyu	2	1	1	4
Luhya	3	0	0	3
Luo	5	3	2	10
Kamba	4	1	0	5
Somali	4	0	1	5
Other	2	1	0	3
Highest education				
No education/preprimary	4	5	1	10
Primary	12	1	3	16
Secondary+	2	0	0	2
Marital status at child birth				
In union	16	0	4	20
Not in union	4	6	0	10
Parity				
1	3	—	—	3
2	4	—	—	4
3	3	—	—	3
4	4	—	—	4
5+	6	—	—	6

The results below are structured around the three thematic areas explored during the study: knowledge on child stunting, views and practices on child feeding, and decision‐making mechanisms on child feeding.

### 3.1 Knowledge and perceptions on child stunting

Respondents were asked to describe symptoms they associated with stunting and its perceived causes. The results show a broad knowledge on child stunting and its symptoms. Regardless of the child's stunting status, there was quasiunanimity among respondents in the two slums that stunted children were usually too small for their age, as two mothers noted.

The child looks small and yet he is older. You will notice from the body, the body does not match the number of years, and if you have such a child, he is sickly and will not want to play with other children. Mother nonstunted child, Viwandani

The child is older but the body frame is small. My youngest is really small. His age mates are much taller …. Mother stunted child, Viwandani

The same statement was echoed by grandmothers from the two slums who associated child stunting symptoms with an imbalance between the age and the height of a child. However, one respondent did not differentiate between stunting and signs of acute malnutrition:

Such child can have swollen legs, pot belly, swollen cheeks; you might think he is fat which is not being fat. You are able to see he is not in good health, which is malnutrition. His hair turns red. You will get the child has sores all over the body. Grandmother, Korogocho

When the causes of stunting were explored, respondents unanimously identified poverty and food insecurity and unbalanced diets as the major reasons. In particular, most of the respondents reported their limited access to food, especially food that is appropriate for feeding young children.

The main problem was lack of food because at times in the morning when she wakes up, there was nothing to eat and she could only get strong tea in the morning, at lunch time maybe you cook white rice with nothing else. Mother stunted child, Viwandani

And this is because this child has been hungry all day and even when the mother comes in the evening she will bring *ugali* and cabbage. The child will then sleep and when morning comes there is not even a cup of tea, the child will then stay hungry till evening again. Grandmother, Korogocho

Poor child health outcome including stunting was seen as a consequence of deprivation and the poor quality of life in the slums, parents' own poor health status, and parents' lack of interaction with their children. For instance, a caregiver's HIV status was related to child stunting in a situation where the affected parent or caregiver essentially prioritizes his or her own health rather than the feeding and health care of the child.

Exploring respondents' views on individual responsibilities regarding child stunting, some fathers were reportedly not fulfilling their role to provide for their families' needs. However, from their own point of view, fathers did not appear to perceive themselves to have a direct responsibility when their child is stunted; the mother or caregiver was identified as the person primarily responsible.

I could tell you … the problem was the food given to the child by the mother. Mostly we, men, are out hustling for the most part of the day, so it is the responsibility of the mother to look after the child. Father, Korogocho

We noted that no reference was made to common practices that may be driven by cultural or religious norms and standards in relation to child feeding. Finally, we also noted that caregivers of stunted children were not always aware that their children were stunted.

### 3.2 Views and practices on child feeding

Respondents' views and actual practices on child feeding were explored from two angles in this study. We first asked specific questions on exclusive breastfeeding in the first 6 months, and then the introduction of complementary feeding was explored. The results show that most of the respondents in the two slums, regardless of the child's stunting status, recognized the importance of exclusive breastfeeding for a newborn but found it hard to sustain up to 6 months. In many cases, there were attempts to exclusively breastfeed in the first 2 or 3 months of child's birth, but for different reasons, mothers opted to stop exclusive breastfeeding before their child reached 6 months. Among the reasons, mothers found it impossible to combine their occupation (some were young studying mothers, housemaids in richer areas of Nairobi, or food sellers on the streets), with exclusive breastfeeding of their child. The findings were observed among both mothers of stunted children and those of nonstunted children.

I used to breastfeed her, but after one month I had to do my KCSE (Kenya Certificate of Secondary Education). Then I started giving her Nan milk (infant formula). Mother nonstunted child, Korogocho

I felt that if I am going to sit here breastfeeding this child, I will suffer even more. So after one week I introduce other food like milk and porridge and any other food that I have found. Even if it is *ugali* and that is how I have been bringing him up. Mother stunted child, Viwandani

However, misinformation about health effects of contraception, often promoted in the postnatal period, was also found.

When I just realized that I was pregnant (five months after birth), I stopped breastfeeding. Mother nonstunted child, Viwandani

I stopped breastfeeding because I was taking family planning contraceptives. I thought the contraceptives were making him sick. Mother stunted child, Viwandani

Being unable to breastfeed exclusively until 6 months was common in the two slums with no difference between mothers of stunted children and those of nonstunted children. Grandmothers, however, were mostly favourable towards exclusive breastfeeding, even though some of them remained unclear on the duration. In many cases, they advised young mothers to comply with the practice and offered their support to take care of babies when mothers were away. However, they seemed not to be cognizant of the complexity of caring for an exclusively breastfed child without having the needed breast milk within easy reach.

Exclusive breastfeeding is good because it is vital in development in the child's brain. Then the milk of the mother helps prevent diseases. The mother's milk is always ready, it needs neither boiling nor cooking, and it has no budget. Grandmother, Korogocho

I think it is important because I noticed it with this baby, when he breastfed for six months exclusively, I have not seen this child disturbing at all. Grandmother, Viwandani

The findings also reveal that there is no standard or informed practice in the study area when breastfeeding is interrupted. Mothers and caregivers usually give the child the types of food that are available depending on their economic capability; a mother of a nonstunted child reported that she would not prepare something different, the child would just eat what is prepared for everybody, because there is no money to prepare weaning foods. In few cases, mothers tried to provide a more structured diet for their newborn.

When I gave birth, after six months I started giving the baby water, fruits, and continued like that, as I introduced other feeds slowly by slowly until the baby was one‐year‐old and able to feed by himself. Mother nonstunted child, Viwandani

Stunted children usually need an appropriate diet in order to improve their nutritional status. During the interviews, mothers, who recognized that their children were stunted, were asked if they thought it was necessary to feed their child differently to help them recover. Affirmative answers were noted from mothers in the two slums. The moment when the child is seen as not growing well and taken frequently to the hospital for care was usually the moment when mothers of stunted children realized the need to take care of their child differently, including changing feeding practices to avoid fatal consequences.

I changed it … so I would give him fruits, milk, bananas, all to boost his appetite. I would make him a mixture with a red fruit and give him. After that he would eat some plain *ugali* followed by some boiled milk. After all these efforts, I would find his health is improving, and he is growing normally. Mother stunted child, Korogocho

However, this finding may not necessarily reflect usual responses to stunting in the slums as a number of mothers provided the food that was available to them as determined by their economic situation. Overall, economic circumstances were more important in determining child‐feeding practices.

Another result is that child‐feeding practices in the slums did not differ by background characteristics, particularly by site or ethnic group. For instance, difficultly to exclusively breastfeed children for 6 months was found among all mothers regardless of their ethnic group.

### 3.3 Decision‐making mechanisms on child feeding

Our interest focused on how decisions on child feeding are taken in the study areas and who the main persons involved are. This information could be the basis of targeted strategies to address child suboptimal feeding in the slums. The findings show that mothers are not the only decision makers on the feeding of their child. Beside health providers, grandmothers were commonly reported to have a key role in that process. Most of the mothers recognized and valued advices from grandmothers, and this was the case for mothers of children who were stunted and not stunted.

When I introduced cow's milk and porridge at six months, my mother is the one who advised me to do that. Mother stunted child, Viwandani

My mother used to advise me on the importance of breastfeeding the child for long. Mother nonstunted child, Korogocho

Grandmothers considered it normal to advise young mothers on how to take care of their babies including child feeding. Most grandmothers proudly assumed that role and justified it by their intention to ensure good health and a better future for their grandchildren. The role of grandmothers in child nutrition in this community is central as it goes beyond the basic advisory tasks. They are also child caregivers and mentors for young mothers as one reported:

It is good for me to sit down with them (young mothers) and teach them how they should feed these children and the types of food that the children should be given, how they should bring up the kids and the importance of taking the children for clinics. Grandmother, Korogocho

However, some grandmothers did not always think their advice was valued. Indeed, their comments implied generational shifts in norms and suggested intergenerational conflict between old and young mothers in this community, as a grandmother reported

These days, you can see how they (young mothers) behave … If you talk to them, they would say ‘what this old woman can teach me?’ I would not be surprised if their children are fed on French fries at night. Grandmother, Korogocho

Looking at the roles of fathers in child feeding, the results show that most of the fathers interviewed indicate that their role is limited to that of providing food, and they have developed strategies to cope with their expected duties. Their role did not extend to feeding children.

## 4 DISCUSSION

This qualitative study aimed to explore the influence of household members on child feeding, in particular, the roles of grandmothers and fathers.

The study shows broad knowledge on child stunting among mothers and grandmothers. Although respondents mostly described stunting as an imbalance between the age and the height of a child, some did not differentiate stunting from acute malnutrition. Other studies from the same setting also found broad knowledge on child stunting among mothers (Kimani‐Murage et al., 2014; Wanjohi et al., 2017). Nonetheless, the fact that some respondents confused stunting with acute malnutrition and that, in this study, some mothers of stunted children did not recognize that their children were stunted raises the question about whether stunting is systematically not recognized. Acute malnutrition can be described as having more obvious signs and symptoms and is therefore more easily recognized. Although, in this study, our respondents could define stunting, a quantitative study would be required to identify if stunting is systematically underrecognized. If so, then specific interventions to raise awareness about stunting in particular would be important.

Fundamentally, irrespective of knowledge, we found that poverty and food insecurity in the slums determine child‐feeding practices and the risk of stunting and recovery from stunting. Many studies have highlighted the levels of deprivation and poor living conditions in these slums and called for interventions to reduce health inequity gaps (Amendah, Buigut, & Mohamed, 2014; Taylor & Goodfellow, 2009; Zulu et al., 2011). The finding that diverse types of solid and liquid food were introduced at early stages (i.e., before 6 months and in one case in the first few weeks of life) depending on the household economic capacity may contribute to explaining the high prevalence of child stunting in the communities. This has also been reported in other studies (Abuya et al., 2012; Kimani‐Murage et al., 2015). Most of the children in these settings are initiated on an adult diet very early in their lives and therefore may not receive the required nutrients from food to prevent growth failure. High poverty levels and poor standards of living may be the main underlying factor that limits households' ability to implement appropriate child feeding. Our finding provides further evidence that interventions and advocacy for more sustainable poverty reduction programmes in these settings are essential.

Importantly, these findings show that mothers are not the sole decision makers regarding the feeding of their children. Grandmothers appear to play key advisory roles in addition to being potential child caregivers. The result confirms the complexity of the dynamics around child nutrition in these settings as reported in previous quantitative study by Faye et al. (2019). The findings are consistent with studies from different settings where senior women were central to the social dynamics and decision making within households and communities (Aubel, 2012; Daglas & Antoniou, 2012; Ekane, 2013). This study confirms that this pattern still exists in urban areas where multigenerational households are still common. However, the effectiveness of grandmothers in influencing child feeding may not be universally successful. This study documented that some grandmothers found that young mothers were not receptive to their advice. In fact, the response of the one grandmother suggested that she was judgmental of the young mother, and this may be an indication of intergenerational conflict between older and younger mothers.

Fathers are described by mothers and grandmothers as providers of food, albeit inadequate, they provide what they can. They are not expected to be involved in decision making around child feeding. Similar results were found in Ethiopia where men mostly focused on food production and provision and were rarely involved in child feeding (Bilal, 2015). However, this could be a barrier to effective child health and development as the involvement of both parents is essential in the feeding and caring of infants and young children. (Guerrero, Chu, Franke, & Kuo, 2016; Mallan et al., 2014). If fathers know more about what weaning children need to eat, they may be more prepared, within the overall limitations of poverty, to provide more appropriate food.

Although our study found that the benefit of breastfeeding is well established as common knowledge in this community, it appeared difficult to implement exclusive breastfeeding as recommended by World Health Organization (2001). Some obstacles are documented in this study including mothers' difficulty to combine their occupation with exclusive breastfeeding of their child. Similar results were found in a previous study where only 2% of the infants in the two settlements were exclusively breastfed for the first 6 months (Kimani‐Murage et al., 2011). It is important to note that most of the grandmothers were supportive of exclusive breastfeeding. These findings call for a more inclusive approach for tackling suboptimal breastfeeding in these communities by targeting both mothers and grandmothers in child nutrition strategies. For instance, initiatives to support mothers to combine breastfeeding and occupations (e.g., studies and work) need to be promoted. One such initiative is human milk banking (APHRC, PATH, & Kenya Ministry of Health, 2016; Arslanoglu et al., 2013; Maingi, Kimiywe, & Iron‐Segev, 2018). This type of initiative could capitalize on grandmothers' willingness to support young mothers, as shown in this study; they could be involved in providing the milk to children when needed. There are some easier wins, for example, misconceptions about using contraception while breastfeeding can be countered by better health communication. However, due to lack of access to health services, those most in need of such information, particularly residents of informal settlements where access to health services is more limited, may not hear this message if only communicated within health care facilities.

Finally, it is important to note some mothers' attempts to change feeding practices of children who were recognized as undernourished in order to help them catch‐up. This indicates a certain awareness in mothers and caregivers of the danger of maintaining stunted children on the same diet that can lead to adverse consequences including child mortality. However, unbalanced change in diet for stunted children may lead to an increased growth velocity with adverse health outcomes in the short or long term (Gerrard & Grant, 2003; Wit & Boersma, 2002). Therefore, further understanding of the dynamics around child catch‐up growth in these deprived settings is needed.

Notwithstanding the importance of these findings, a few limitations need to be highlighted. We did not relate the views of all respondents from the same household. Also, the fact that there were no differences in views and feeding practices between households of stunted children and those of nonstunted children may be related to the small number of households interviewed in this study. A bigger sample size may have contributed to enriching the narratives and provide a better understanding of child‐feeding practices and decision‐making mechanisms within each household. In addition, some mothers of stunted children did not recognize that their child was stunted, and this limited interviewers' ability to ask probing questions to better understand feeding practices for stunted children. Finally, having used a qualitative approach, the study did not allow for a large sample size, and therefore, findings cannot be generalized.

## 5 CONCLUSION

The study explored mothers' perceptions and practices on child feeding and highlighted the importance of other family members in decision making related to child health issues in the two Nairobi informal settlements: Korogocho and Viwandani. The study finds that grandmothers have central roles in child nutrition as they are key advisors to younger mothers. As such, they are important targets for child health interventions and should be included in strategies aiming to reduce child malnutrition in these settings. The findings suggest these interventions should focus on (a) increasing mothers' knowledge on child nutrition including removing the misconceptions around breastfeeding and pregnancy or use of contraceptives; (b) prioritizing interventions that support mothers to combine breastfeeding and other occupations, through human milk banking, and other baby‐friendly initiatives; (c) promoting optimal child‐feeding practices, in particular, when exclusive breastfeeding ends; and (d) reinforcing interventions for more sustainable poverty reduction programmes in these settings.
